# Supplementary material for: Complexome profiling of the Chlamydomonas psb28 mutant reveals TEF5 as an early PSII assembly factor
Source: Plant Cell. 2025 Mar 17;37(6):koaf055. doi: 10.1093/plcell/koaf055 (PMC12164590; doi:10.1093/plcell/koaf055)
Supplement: koaf055_Supplementary_Data [file koaf055_supplementary_data.zip › psb28_interactive.zip!/plots/intensity/qualityPlots/peptides/AST4_Cre06.g257950.t1.2.html]

### Cre06.g257950.t1.2

Trivial name: AST4  
SeqCoverage [median]: 38.20  
Mass: 47.74 kDa  
  
 

| Sequence | MissedCleavage | Mass [Da] | wtIntensity | muIntensity | Score |
| --- | --- | --- | --- | --- | --- |
| AIARPMYSNPPLHGALLVTK | 1 | 2148.19 | 518.93 | 0.00 | 53.28 |
| DAGVEFAK | 0 | 835.41 | 12697.67 | 27980.49 | 133.60 |
| DASAVESQLK | 0 | 1046.52 | 24582.25 | 43737.26 | 186.67 |
| DFISESVK | 0 | 923.46 | 0.00 | 2287.41 | 66.80 |
| FLADTNPIK | 0 | 1017.55 | 8979.88 | 22062.20 | 155.07 |
| GLDMDGFLEDIK | 0 | 1351.63 | 1944.90 | 6938.96 | 151.15 |
| IFLADGHR | 0 | 927.49 | 0.00 | 1035.12 | 70.00 |
| ILQDNDLK | 0 | 957.51 | 21606.46 | 35370.63 | 139.88 |
| ISMAGVNTK | 0 | 919.48 | 14774.19 | 14609.10 | 124.92 |
| LAEAMHAVTSGKL | 1 | 1326.70 | 20645.80 | 45191.01 | 110.92 |
| LAVSQSYAK | 0 | 965.52 | 31376.95 | 51290.20 | 196.99 |
| LFADFQK | 0 | 867.45 | 301.97 | 0.00 | 89.70 |
| MNLGVGAYRDDNGQPVVLDAVR | 1 | 2358.18 | 0.00 | 8444.89 | 85.42 |
| NMGLYGQR | 0 | 937.44 | 3207.75 | 2812.84 | 125.01 |
| PMYSNPPLHGALLVTK | 0 | 1736.93 | 0.00 | 7064.66 | 92.98 |
| VAGSHFMEYLPIGGLR | 0 | 1745.89 | 947.81 | 12086.84 | 64.67 |
|  |  |  | 141584.6 | 280911.6 |  |
